# Deep learning-based simultaneous registration and unsupervised non-correspondence segmentation of medical images with pathologies

**DOI:** 10.1007/s11548-022-02577-4

**Published:** 2022-03-03

**Authors:** Julia Andresen, Timo Kepp, Jan Ehrhardt, Claus von der Burchard, Johann Roider, Heinz Handels

**Affiliations:** 1 Institute of Medical Informatics, University of Lübeck, Ratzeburger Allee 160, 23562 Lübeck, Germany; 2 German Research Center for Artificial Intelligence, Lübeck, Germany; 3 Department of Ophthalmology, Christian-Albrechts-University of Kiel, Kiel, Germany

**Keywords:** Image registration, Non-correspondence detection, Pathology segmentation, Convolutional neural network, Optical coherence tomography

## Abstract

**Purpose:**

The registration of medical images often suffers from missing correspondences due to inter-patient variations, pathologies and their progression leading to implausible deformations that cause misregistrations and might eliminate valuable information. Detecting non-corresponding regions simultaneously with the registration process helps generating better deformations and has been investigated thoroughly with classical iterative frameworks but rarely with deep learning-based methods.

**Methods:**

We present the joint non-correspondence segmentation and image registration network (NCR-Net), a convolutional neural network (CNN) trained on a Mumford–Shah-like functional, transferring the classical approach to the field of deep learning. NCR-Net consists of one encoding and two decoding parts allowing the network to simultaneously generate diffeomorphic deformations and segment non-correspondences. The loss function is composed of a masked image distance measure and regularization of deformation field and segmentation output. Additionally, anatomical labels are used for weak supervision of the registration task. No manual segmentations of non-correspondences are required.

**Results:**

The proposed network is evaluated on the publicly available LPBA40 dataset with artificially added stroke lesions and a longitudinal optical coherence tomography (OCT) dataset of patients with age-related macular degeneration. The LPBA40 data are used to quantitatively assess the segmentation performance of the network, and it is shown qualitatively that NCR-Net can be used for the unsupervised segmentation of pathologies in OCT images. Furthermore, NCR-Net is compared to a registration-only network and state-of-the-art registration algorithms showing that NCR-Net achieves competitive performance and superior robustness to non-correspondences.

**Conclusion:**

NCR-Net, a CNN for simultaneous image registration and unsupervised non-correspondence segmentation, is presented. Experimental results show the network’s ability to segment non-correspondence regions in an unsupervised manner and its robust registration performance even in the presence of large pathologies.

## Introduction

Image registration describes the process of finding an optimal deformation that transforms one image such that it is similar to another image and corresponding image structures align spatially. Typically this is done by minimizing a loss functional composed of an image distance measure and a regularizer that smooths the deformation field. Such methods are based on the assumption that for every pixel in the moving image there exists a corresponding pixel in the fixed image. In medical images, this assumption often does not hold due to pathologies either changing over time or being present in only one of the images. Registering pathology images directly can lead to huge registration errors since intensity differences are erroneously accounted for by image deformations.

Several approaches exist to handle non-corresponding regions in image registration. One solution is cost function masking or weighting. The easiest approach is to first segment non-corresponding regions and then use the segmentation to mask the image distance measure during registration [1, 2], but this requires that non-corresponding regions are known before registration. Especially when registering images containing evolving pathologies, the generation of a ground truth of non-correspondences is often not feasible.

Works detecting non-correspondences during the optimization process, e.g. [3–8], overcome this limitation. Chen et al. detect non-corresponding regions based on outlier detection in the distance measure combined with regularization [3]. Ou et al. introduce the mutual-saliency weighting which is based on an automatic estimation of the matching uniqueness between voxel pairs after deformation [4]. Krüger et al. estimate correspondence probabilities between sparse image representations to weight the image distance during registration. The correspondence probabilities are further used to segment pathologies in medical images [5, 6]. Metamorphoses models such as [9–12] model both spatial deformations and appearance changes to match moving and fixed image. These have extensively been used to model evolving processes.

Further approaches that handle the registration of pathological to healthy images try to transform pathological images such that they appear healthy or to introduce pathologies in healthy images [13–15]. In [13], a tumor growth model is implemented that introduces artificial tumors in brain atlases which are then registered to the respective MRIs, whereas [14, 15] estimate healthy versions of images containing pathologies using low-rank plus sparse image decomposition.

Common to all these works is that the optimal deformation is found using iterative optimization schemes. Thus, they are time-consuming. Recent image registration algorithms are often based on convolutional neural networks (CNNs) achieving state-of-the-art performance while greatly reducing computation time in comparison with classical image registration algorithms, e.g. [16–23]. The networks are either trained supervised using given deformations as ground truth [16–18] or unsupervised based on image distance measures and regularization as in classical image registration algorithms [19–21]. Weak supervision based on manual segmentations may be introduced by additional loss terms giving feedback on the overlap of corresponding structures [22, 23]. For an overview of existing deep learning-based image registration methods, refer to [24–26].

The literature on joint image registration and (unsupervised) non-correspondence estimation with deep learning methods, however, is still scarce. Unsupervised methods to estimate registration uncertainty can for example be found in [27, 28]. These are based on Monte Carlo dropout and thus require several runs during inference. Zhou et al. [29] present a CNN to establish visual correspondence across different object instances. The network outputs a flow field from source to target image and a probability map indicating pixel correspondences. Network training, however, relies on synthetic ground truth.

Sedghi et al. [30] propose a classifier network that patch-wise predicts class probabilities for either *registered*, *unrelated* or 18 different transformations. The deformation for unrelated patches is set to zero, regularizing the deformation in non-correspondent regions. This configuration allows to directly estimate non-correspondences, but the method relies on an iterative scheme and is not applicable on voxel level.

**Fig. 1:**
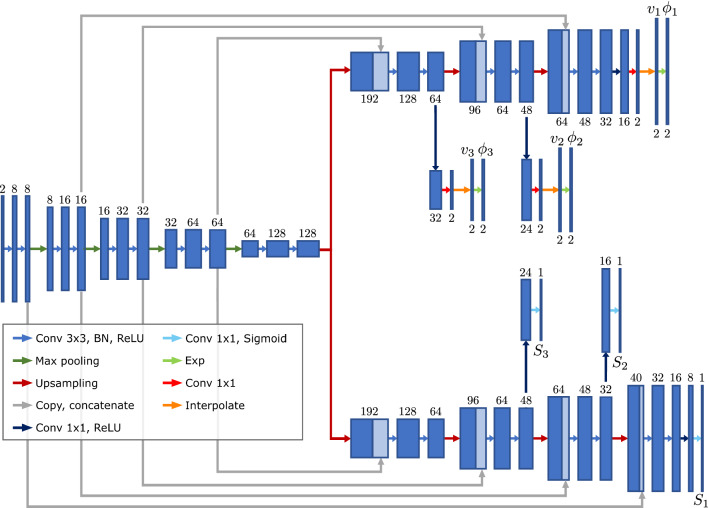
Architecture of NCR-Net. Input to the network are two affinely pre-aligned pathology images. Output of the registration branch are three diffeomorphic deformations $\phi _1$, $\phi _2$ and $\phi _3$ that transform the moving image to the fixed image at different resolution levels. The segmentation branch outputs the segmentations $S_1$, $S_2$ and $S_3$ of non-corresponding regions for the three resolution levels. Numbers above or below the blue boxes indicate the number of feature maps, and BN stands for batch normalization

In this paper, we train a CNN to densely register medical images in one-shot and simultaneously segment regions of non-correspondence. Using a Mumford–Shah-like functional as loss function, we train the joint non-correspondence segmentation and registration network (NCR-Net) to perform intra-patient registration of retinal optical coherence tomography (OCT) image slices (B-scans) and inter-patient registration of magnetic resonance images (MRIs) with phantom lesions. The loss function is based on a masked image distance measure combined with several regularization terms, smoothing the deformation field and favoring small and smooth segmentations of non-corresponding image regions. To the best of our knowledge, this is the first work adapting the variational joint registration and segmentation approach [3] for CNN training. We extend the approach introducing a two-step training procedure that allows to better disentangle spatial deformations and cost function masking. Our NCR-Net achieves a performance competitive to state-of-the-art registration methods while having the great advantage of generating non-correspondence maps that allow for unsupervised segmentation of pathologies, inter- and intra-patient variations and areas of disease progression. The main contributions of this work are:

- A new network architecture for joint image registration and unsupervised non-correspondence segmentation
- Introduction of a two-step training scheme to train the proposed CNN
- Proof that NCR-Net may be used for unsupervised lesion segmentation and that it can compete with state-of-the-art registration frameworks

## Materials and methods

### Network architecture

The proposed network consists of an encoder and two decoders whose architecture is inspired by the U-Net [31]. Together, they form a y-shaped network architecture with both decoders being connected to the encoding branch via skip connections as shown in Fig. 1. The numbers of filters used in the network were determined empirically by varying the number of output features in the first convolutional block between four and 16 and setting the number of filters in the following blocks dependent on this number. Input to the network are the moving image $\mathrm {M}:\varOmega \xrightarrow {} \mathbb {R}$ and the fixed image $\mathrm {F}:\varOmega \xrightarrow {} \mathbb {R}$. The first decoder represents the registration branch that outputs three diffeomorphic deformation fields $\phi _1$, $\phi _2$ and $\phi _3$ warping the moving image to match the fixed image at three levels of resolution [18, 23]. The registration branch first generates vector fields $v_i$ which are interpreted as stationary velocities and then applies an exponentiation layer to generate diffeomorphic transformations $\phi _i$ as described in [32]. We use the implementation provided in [33]. The second decoder outputs segmentations $S_1$, $S_2$ and $S_3$ of non-corresponding regions for the three resolution levels.

### Loss functions and training procedure

For the registration of pathological images, we seek a deformation $\phi :\mathbb {R} \xrightarrow {} \mathbb {R}^d$ that transforms the moving image such that $\mathrm {M}(\phi (x))$ is similar to $\mathrm {F}(x)$ for every pixel position *x* in $\varOmega \setminus \mathcal {S}$ with $\mathcal {S}$ being the non-corresponding region. A Mumford–Shah-like functional

1 $$\begin{aligned} \mathcal {L}_{\text {MS}}(\theta ; \mathrm {M}, \mathrm {F})&= \sum _{x \in \varOmega } \big ( 1 - S \big ) \cdot \mathcal {D} \big [ \mathrm {F}, \phi \circ \mathrm {M} \big ]\nonumber \\&\quad + \mathcal {R}_\phi + \mathcal {R}_N + \lambda \, \mathrm {Dice} \big [ \mathrm {S_F}, \phi \circ \mathrm {S_M} \big ] \nonumber \\ \mathcal {R}_\phi&= \alpha \, \Vert \nabla v \Vert _2^2 \nonumber \\ \mathcal {R}_N&= \beta \, S + \gamma \, \mathrm {tanh} \big ( \Vert \nabla S \Vert _2 \big ) \end{aligned}$$

is used as loss function for the training of NCR-Net to optimize the network parameters $\theta$. The image distance measure $\mathcal {D}$ is masked with the segmentation output *S* of the network to assure that it is evaluated on corresponding image regions only. The second term $\mathcal {R}_\phi$ regularizes the deformation field $\phi =\exp (v)$ by enforcing smoothness of the stationary velocity field *v*. The loss components of $\mathcal {R}_N$ approximate the volume and the perimeter of the segmentation *S* thus favoring small segmentations with smooth boundaries. This part of the loss function is similar to the functional used in [3] for a classical image registration approach. The flexibility of the CNN-based approach now allows any further loss components to be added. To stabilize training and introduce weak supervision to the registration task, we add an additional term to the loss function that is based on the overlap of given segmentations of the moving and the fixed image. These segmentations do not delineate pathologies but large anatomical regions in the images (e.g., the brain in MR images of the head or the retina in OCT images) and shall be defined by $\mathrm {S_F}$ and $\mathrm {S_M}$ for fixed and moving image, respectively.

The highly entangled character of the loss function might cause network training to converge to local minima, where spatial misalignments are masked out instead of being compensated for by the registration branch of the network or where non-correspondences lead to registration errors instead of being masked out. We therefore propose to use a two-step training scheme that first pre-trains encoder and registration branch using

2 $$\begin{aligned} \mathcal {L}_{\text {Reg}}(\theta ; \mathrm {M}, \mathrm {F}) = \sum _{x \in \varOmega } \mathcal {D} \big [ \mathrm {F}, \phi \circ \mathrm {M} \big ] + \mathcal {R}_\phi + \lambda \, \mathrm {Dice} \big [ \mathrm {S_F}, \phi \circ \mathrm {S_M} \big ] \end{aligned}$$

as loss function and only then use $\mathcal {L}_{\text {MS}}$ to train the entire network. The loss functions (1) and (2) are evaluated on all three resolution levels, and a weighted sum of the three losses is calculated to give the final loss. Let $\mathcal {L}^i(\theta ; \mathrm {M}, \mathrm {F})$ be the loss function (1) or (2) evaluated on the *i*-th resolution level, then

3 $$\begin{aligned} \mathcal {L}(\theta ; \mathrm {M}, \mathrm {F}) = \omega _1 \, \mathcal {L}^1 + \omega _2 \, \mathcal {L}^2 + \omega _3 \, \mathcal {L}^3, \end{aligned}$$

with $\omega _1\!>\!\omega _2\!>\!\omega _3$ and $\omega _1\!+\!\omega _2\!+\!\omega _3=1$, defines the final loss function giving higher weight to finer resolution levels [23].

For OCT registration, we use the same parameters as done in [23], namely $\omega _1\!=\!0.5$, $\omega _2\!=\!0.3$ and $\omega _3\!=\!0.2$. For MRI registration, we found $\omega _1\!=\!0.7$, $\omega _2\!=\!0.2$ and $\omega _3\!=\!0.1$ to give better results. The proposed network is implemented in the PyTorch framework and trained for 500 epochs with an initial learning rate of 1e$^{-4}$ and Adam optimization. For the first 250 epochs, $\mathcal {L}_\mathrm {Reg}$ is used as loss function and for the last 250 epochs the network is trained with $\mathcal {L}_\mathrm {MS}$. The weighting parameters $\alpha$, $\beta$, $\gamma$ and $\lambda$ are found empirically and set to 0.8, $2.4\mathrm {e}^{-7}$, $1.2\mathrm {e}^{-7}$ and 0.4 for the OCT experiments, respectively. For the MRI experiments, parameters are set to 2, $1.3\mathrm {e}^{-6}$, $5.1\mathrm {e}^{-7}$ and 1 if not stated otherwise. For each experiment, we perform fivefold cross-validation.

## Experiments

In the following, NCR-Net is used to perform two different registration and non-correspondence segmentation tasks:

1. Intra-patient registration of OCT images of patients suffering from neovascular age-related macular degeneration (AMD)
2. Inter-patient registration of MRIs with phantom lesions added to the fixed image.

Since the given OCT volumes have a large inter B-scan distance, a 3D registration does not seem plausible here and we apply NCR-Net to 2D B-scans separately. Unfortunately, there are no ground truth segmentations of retinal fluids or non-correspondent regions given for the OCT dataset which makes quantitative evaluation of the non-correspondence segmentations impossible. We therefore quantitatively validate the 2D approach based on the second task that naturally delivers ground truth lesion segmentations since lesions are introduced artificially. We perform the following experiments:

- Ablation Studies:

- Weakly supervised vs. fully unsupervised registration (OCT)
- Performance with vs. without non-correspondence detection (MR, OCT)
- Two-step training scheme vs. full loss function throughout all epochs (OCT)

- Unsupervised and weakly supervised pathology segmentation

- Weakly supervised segmentation of phantom stroke lesions (MR)
- Unsupervised segmentation of retinal fluids (OCT)

- Comparison to state-of-the-art image registration algorithms (MR).

Finally, we extend NCR-Net to 3D, compare its performance to another deep learning-based registration approach and use it for lesion segmentation on 3D MRIs.

### Data

#### OCT images of AMD patients

The dataset used for OCT registration consists of 709 OCT volumes from 41 AMD patients monitored for several years. Follow-up times range from 32.5 months to 82.8 months. The OCT images were taken with eye-tracking on a Heidelberg Spectralis system and provided by the Department of Ophthalmology in Kiel. The images have a size of $496\!\times \!512\!\times \!25\;$pixels with a field of view size of $2\!\times \!6\!\times \!6\;\mathrm {mm}^3$. For each volume the inner limiting membrane (ILM), the retinal pigment epithelium (RPE) and the Bruch’s membrane (BM) were manually delineated by a medical expert. Out of these 709 OCT images 193 image pairs (9650 B-scans) from 40 patients are selected for which the acquisition times of first and second image are no longer apart than five months. For intra-patient OCT registration the baseline image is defined as moving and the follow-up image as fixed image. Furthermore, we define $\mathrm {S_M}$ and $\mathrm {S_F}$ as the given retina segmentation of baseline and follow-up images, respectively. The retina is defined as the area between ILM and BM. A spatial transformer network is trained to rigidly pre-align Gaussian-smoothed OCT B-scans using the mean squared error (MSE) as loss function. The pre-aligned images are cropped to the central 384 A-scans to assure the given segmentations span the whole image width. The cropped images serve as inputs of NCR-Net. As distance measure for the loss function of NCR-Net we again use MSE. Online data augmentation is performed by randomly rotating between $-7$% and $+7$%, vertically shifting the moving image (by at most one quarter of the image height) and flipping both images horizontally. The data are split on patient-level into training (32 patients) and test (8 patients) data.

#### LPBA40 image slices with phantom lesions

The LPBA40 dataset consists of 40 whole-head MRI volumes and manual segmentations of 56 anatomical regions [34, 35]. Image size is $181\!\times \!217\!\times \!181$ voxels with an isotropic spacing of $1\ \text {mm}$. In previous work [5, 6] we inserted four different stroke lesions from the ISLES dataset [36] into the images for evaluation purposes. The artificially inserted lesions (L1, L2, L3, L4) differ in size and appearance with two of the four lesions being huge and corrupting large areas of the images. Each of the four lesions is introduced into each MRI separately, leading to five versions of each image (original plus four corrupted images containing lesion L1, L2, L3 and L4, respectively). As the lesions are introduced into the images artificially, ground truth segmentations of the pathologies are available that we use to evaluate the segmentation performance of NCR-Net.

Again, the data are split on patient level into training (32 patients) and test datasets (8 patients). The CNN is trained performing pairwise registration of each lesion-free image to all images available from the remaining 31 patients. Here, $\mathrm {S_M}$ and $\mathrm {S_F}$ are defined as automatic brain segmentations resulting from skull-stripping. Normalized cross correlation is used as image distance measure for LPBA40 registration. We perform online data augmentation by randomly rotating one of the images between $-3^\circ$ and $+3^\circ$ or both images between $-8^\circ$ and $+8^\circ$ and shifting them by a maximum of 4 pixels in each direction. Additionally, images are randomly flipped in the horizontal axis and Gaussian noise is added to the images. For 3D registration we downsample the image volumes to a resolution of $96\!\times \!96\!\times \!112$ voxels and in 2D we register the central transversal MR slices.

### Ablation studies

In this section, we analyze different aspects of our proposed NCR-Net. First, we train NCR-Net with and without the described weak supervision of the registration task. That is, we train the network using (1) as loss function once with and once without the Dice loss component. Second, we analyze the influence of the non-correspondence segmentation on the registration performance of the network comparing NCR-Net to a registration-only network called RegNet. RegNet is identical to NCR-Net except that it has no segmentation branch. To train RegNet we use (2) as loss function. Finally, we train NCR-Net with the proposed two-step training scheme, initially only updating the registration part of the network and later the entire network. In summary, this means that four CNNs are trained:

1. RegNet: Pure registration network (no non-correspondence segmentation)
2. NCR-Net$_\mathrm{b}$: Baseline NCR-Net trained using (1)
3. NCR-Net$_\mathrm{u}$: Fully unsupervised NCR-Net trained using (1) without Dice loss
4. NCR-Net$_\mathrm{s}$: NCR-Net trained with the proposed two-step training scheme.

**Table 1:** OCT registration results: average symmetric surface (ASSD) and Hausdorff distances (HD) of ILM, RPE and BM before and after registration with RegNet, baseline NCR-Net (NCR-Net$_\mathrm {b}$), unsupervised NCR-Net (NCR-Net$_\mathrm {u}$) and two-step training NCR-Net (NCR-Net$_\mathrm {s}$)

	ILM		RPE		BM	
Method	ASSD	HD	ASSD	HD	ASSD	HD
w/o reg.	6.825 ($\pm 7.507$)	15.152 ($\pm 14.008$)	6.269 ($\pm 7.281$)	13.936 ($\pm 12.179$)	6.642 ($\pm 8.423$)	12.220 ($\pm 12.424$)
RegNet	**0.852** ($\pm 1.787$)	**3.483** ($\pm 5.983$)	**1.861** ($\pm 1.819$)	**7.313** ($\pm 6.023$)	**2.796** ($\pm 4.168$)	**8.478** ($\pm 9.367$)
NCR-Net$_\mathrm {b}$	1.072 ($\pm 2.952$)	4.369 ($\pm 8.517$)	2.198 ($\pm 3.252$)	8.213 (±7.993)	3.104 ($\pm 5.115$)	9.135 ($\pm 10.459$)
NCR-Net$_\mathrm {u}$	1.086 ($\pm 2.998$)	4.416 ($\pm 8.649$)	2.192 ($\pm 3.272$)	8.162 ($\pm 7.906$)	3.095 ($\pm 5.128$)	9.100 ($\pm 10.414$)
NCR-Net$_\mathrm {s}$	0.927$^*$ ($\pm 2.174$)	3.765$^*$ ($\pm 6.898$)	2.100$^*$ ($\pm 2.741$)	7.888$^*$ ($\pm 7.102$)	3.021$^*$ ($\pm 4.759$)	8.951 ($\pm 10.003$)

Significantly best results are presented in bold font. The NCR-Net version, which significantly outperforms the other NCR-Net versions, is marked with $^*$. All tests are performed using one-sided Wilcoxon signed rank tests with significance level 0.05

**Fig. 2:**
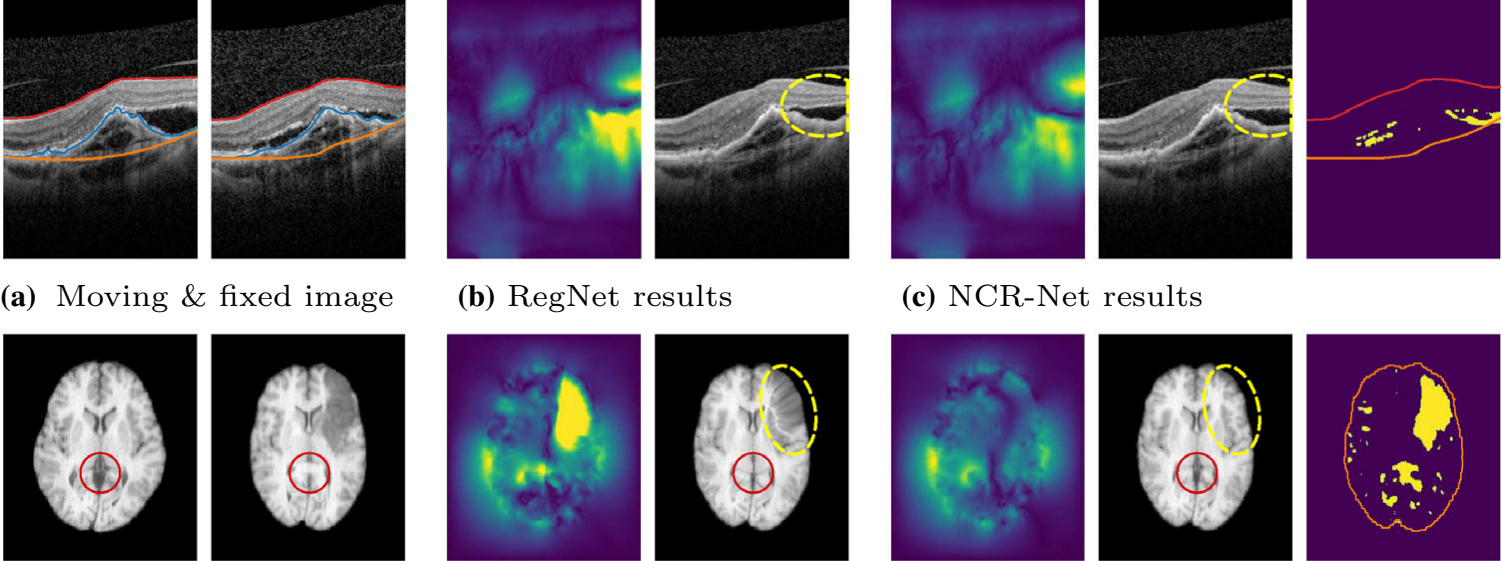
Results of RegNet and NCR-Net$_\mathrm {s}$ for the registration of OCT B-scans (upper row) and MRI slices (lower row). ILM, RPE and BM are marked in the OCT images with red, blue and orange lines, respectively. In (**b**) and (**c**) deformation fields, deformed moving images and, if applicable, segmentations of non-correspondent image regions are shown. The non-correspondence segmentation of NCR-Net allows for more plausible deformations in pathological (yellow dashed ovals) and non-correspondent (red circles) areas

In Table 1, results of all four networks are reported for OCT image registration. Mean Hausdorff and average symmetric surface distances of ILM, RPE and BM are given before and after registration with different CNNs. Results show that the registration performance is similar for NCR-Net$_\mathrm {b}$ and NCR-Net$_\mathrm {u}$ despite the unsupervised training of NCR-Net$_\mathrm {u}$. When using one-sided Wilcoxon signed rank tests (significance level 0.05) to compare results of the two network versions, only the performance difference for the ILM results is significant. The proposed method may thus also be used for datasets without any given annotations while maintaining good registration performances.

Table 1 also shows that the two-step training procedure significantly improves the registration performance for the OCT data. As hypothesized in “Loss functions and training procedure“ section, pre-training the network on the registration task can actually help to better disentangle the registration and non-correspondence segmentation tasks. All upcoming experiments are therefore performed using the two-step training procedure.

The registration-only network slightly but significantly outperforms NCR-Net for the OCT data. Still, RegNet tends to give implausible deformations in pathological image areas as shown in Fig. 2. This behavior is even more apparent for MRI registration as the phantom stroke lesions L1 and L4 are very large. In Fig, 2 it can be seen how lesion L1 impairs registration performance of RegNet and how a healthy structure that is only visible in one of the two MR images (red circles in Fig, 2) is eliminated by the registration-only network that tries to compensate intensity differences with implausible deformations. NCR-Net manages to plausibly deform the pathological area and to retain the healthy structure thanks to the masking of the image distance measure. In Table 2 quantitative results comparing RegNet and NCR-Net for MRI registration are given. Since voxel-level segmentations are available for the MRIs we report mean Jaccard indices of the 19 labels that are present in all 2D slices used. Again it can be seen how NCR-Net benefits from the non-correspondence segmentation leading to significantly better performance for lesions L1 and L4.

**Table 2:** MRI registration results: The mean Jaccard indices of 19 labeled anatomical regions are reported before and after registration of LPBA40 with four phantom stroke lesions (lesion types L1–L4)

Method	No Lesion	L1	L2	L3	L4
w/o reg.	0.480 (0.063)	0.480 ($\pm 0.063$)	0.480 ($\pm 0.063$)	0.480 ($\pm 0.063$)	0.480 ($\pm 0.063$)
RegNet	**0.620** (0.0335)	0.509 ($\pm 0.032$)	0.610 ($\pm 0.033$)	**0.620** ($\pm 0.033$)	0.528 ($\pm 0.034$)
NCR-Net	0.619 (0.034)	**0.600** ($\pm 0.036$)	**0.619** ($\pm 0.034$)	0.619 ($\pm 0.0340$)	**0.595** ($\pm 0.035$)

Significantly best results are presented in bold font (one-sided Wilcoxon signed rank test with significance level 0.05)

**Fig. 3:**
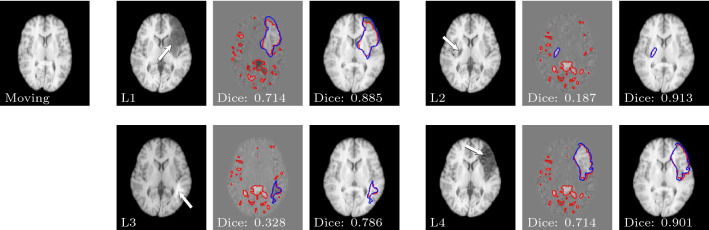
Registration and segmentation results of NCR-Net for four different lesions added to the LPBA40 dataset. The second and fifth columns show the fixed image with the artificially inserted lesions. In the third and sixth columns the difference image between fixed and warped moving image is shown with the segmentation output of the network overlaid in red and the ground truth lesion segmentation in blue. The fourth and seventh columns show the warped moving image together with the lesion segmentation after region growing in red and the ground truth segmentation in blue

### Unsupervised and weakly supervised pathology segmentation

The non-correspondence maps resulting from NCR-Net may be used to segment pathologies in a weakly supervised or even fully unsupervised manner. We will evaluate the segmentation capacity of NCR-Net quantitatively based on the LPBA40 dataset with phantom stroke lesions and show exemplary qualitative results for unsupervised fluid segmentation in OCT images.

**Table 3:** Mean DSCs between ground truth lesion segmentations and segmentations given by NCR-Net binarized by simple thresholding with threshold 0.5 (TH) and segmentations resulting from performing region growing on the network’s output (RG)

Method	L1	L2	L3	L4
ProbReg, TH	$0.693\;(\pm \; 0.174)$	$\mathbf{0}.278 \;(\pm \; 0.174)$	$0.126 \;(\pm \; 0.092)$	–
NCR-Net, TH	$\mathbf{0}.742 \;(\pm \; 0.067)$	$0.225 \;(\pm \; 0.086)$	$\mathbf{0}.362 \;(\pm \; 0.107)$	$0.737\;(\pm \; 0.067)$
ProbReg, RG	$0.865\;(\pm \; 0.111)$	$0.629\;(\pm \; 0.172)$	$\mathbf{0}.754 \;(\pm \; 0.156)$	–
NCR-Net, RG	$\mathbf{0}.871 \;(\pm \; 0.049)$	$\mathbf{0}.870 \;(\pm \; 0.040)$	$0.630\;(\pm \; 0.157)$	$0.880\;(\pm \; 0.041)$

For comparison the results by Krüger et al. [6] are shown who use thresholding and region growing on correspondence probability maps to segment lesions. Best results presented in bold font

#### Weakly supervised segmentation of stroke lesions in MR images

As shown in Fig. 2, the segmentations given by NCR-Net for inter-patient MRI registration do not only contain pathologies but also normal inter-patient variations are segmented. To quantitatively evaluate the network’s lesion segmentation performance we therefore postprocess the non-correspondence maps given by NCR-Net performing region growing based on the assumption that a correct seed point inside the lesions may be defined. We determine such seed points by calculating the overlap between segmentation output and ground truth and randomly selecting one voxel inside the overlap region. The resulting lesion segmentations are compared to the ground truth lesion masks by calculating the mean Dice similarity coefficients (DSCs) between the segmentations. We show exemplary results in Fig. 3 and report the results averaged over all images in Table 3. For comparison we cite the results achieved by Krüger et al., who estimate correspondence probability maps and use these maps to generate lesions segmentations. For this purpose they perform thresholding and region growing with two different thresholds and keep the best-performing method per image [6].

**Fig. 4:**
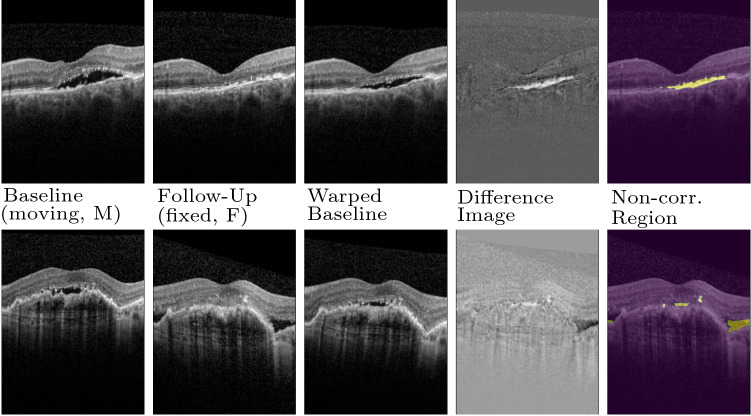
Registration and non-correspondence segmentation results of NCR-Net for two AMD patients. Each row corresponds to one patient. The last column shows the follow-up image with the segmentation of non-corresponding regions overlaid

The results show that simple thresholding on the segmentation output of NCR-Net leads to comparable or better segmentation results than thresholding performed on correspondence probability maps. Using region growing NCR-Net again outperforms the competitive method for two out of three lesions used in [6]. For lesion L4 NCR-Net also achieves good results with a mean Dice score of 0.880. All in all, NCR-Net achieves high overlap with the ground truth lesion segmentations for all four lesion types showing its potential for unsupervised (thresholding on segmentation output) or weakly supervised (e.g., region growing with seed inside lesion) lesion segmentation quantitatively.

#### Unsupervised fluid segmentation in OCT images

In Fig. 4, exemplary OCT image registration and non-correspondence segmentation results of NCR-Net are shown for two AMD patients. Since we are performing intra-patient registration over close timepoints structural differences between the two images are solely due to lesions either progressing from baseline to follow-up or being present for only one of the two time-points. As shown in the figure, the network training based on outlier detection in the image distance measure combined with regularization enables the network to delineate areas of disease progression. Even though our method is fully unsupervised NCR-Net produces sharp and very detailed pathology segmentations, a huge advantage compared to other unsupervised segmentation approaches such as [37]. This shows great potential of our network to be used for the monitoring of progressive diseases such as AMD without the need for expensive manual segmentations.

**Fig. 5:**
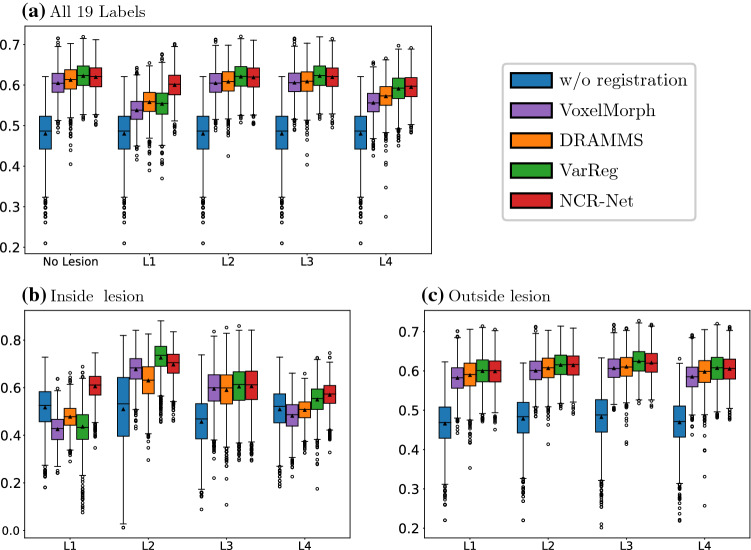
Jaccard indices of 19 labeled anatomical regions before (blue) and after 2D registration with VoxelMorph (purple), DRAMMS (orange), VarReg (green) and NCR-Net (red). In **a** results for all 19 labels are reported, while in **b** only those labels are considered that overlap with the lesion. In **c** Jaccard indices for labels outside of the lesions are shown

**Table 4:** Registration results of NCR-Net for 3D MRI registration compared to VoxelMorph. The mean Jaccard indices of 56 labeled anatomical regions are reported

Method	No Lesion	L1	L2	L3	L4
w/o reg.	0.373 (± 0.048)	0.373 (± 0.048)	0.373 (± 0.048)	0.373 (± 0.048)	0.373 (± 0.048)
VoxelMorph 3D	0.480 (± 0.056)	0.466 (± 0.052)	0.480 (± 0.056)	0.474 (± 0.053)	0.464 (± 0.052)
NCR-Net 3D	**0.489**$^*$ (± 0.033)	**0.486**$^*$ (± 0.032)	**0.487**$^*$ (± 0.033)	**0.476** (± 0.031)	**0.486**$^*$ (± 0.032)

Best results are presented in bold font and $^*$ mark significantly higher results according to a one-sided Wilcoxon signed rank test with significance level 0.05

### Comparison to state-of-the-art registration algorithms

For comparison of our framework to state-of-the art registration algorithms we use the variational registration algorithm (VarReg) by Ehrhardt et al. [38], the deformable registration via attribute matching and mutual-saliency weighting (DRAMMS) by Ou et al. [4, 39] and the diffeomorphic extension of the deep learning-based approach VoxelMorph by Balakrishnan et al. [20, 40] as competitive algorithms. Both VarReg and Dramms are among the best performing algorithms on the LPBA40 dataset and open-source [38, 39]. The mutual-saliency weighting of DRAMMS serves to reduce the impairment done by non-corresponding regions similar to our masking of the distance measure. We use the default parameters of the DRAMMS algorithm [6, 39] and VarReg is performed with curvature regularization and the normalized cross correlation distance measure. The VoxelMorph network is trained with the same learning rate schedule as NCR-Net using (2) as loss function. The network architecture is the same as in the original VoxelMorph papers [20, 40].

The results for MRI registration with different methods are shown in Fig. 5. As done in [6], we measure the registration performance by calculating the average Jaccard index of anatomical labels once for all 19 given labels and once for the labels inside and outside of the inserted lesions. The results show that the proposed CNN can compete with VoxelMorph, DRAMMS and VarReg. Especially for very large lesions (L1 and L4), NCR-Net manages to produce plausible deformations while the performance of competitive methods drops substantially. For images containing no or small lesions (L2 and L3) NCR-Net outperforms VoxelMorph and DRAMMS while results for VarReg are slightly yet significantly (one-sided Wilcoxon signed rank test with significance level 0.05) better than the results of NCR-Net. Still, VarReg lacks the advantage of producing non-correspondence maps and is less robust to non-correspondences since it is not tailored to handle non-correspondent image regions. While the performance of VarReg consequently drops for large lesions, NCR-Net performs similar for all lesion types considered. This shows that NCR-Net achieves good registration results independently of wide varieties of lesions while being especially useful for the registration of images containing large pathologies that lead to registration errors in image registration methods that do not account for non-correspondences such as VoxelMorph or VarReg.

### 3D MRI registration

In this experiment, we extend NCR-Net to 3D and perform pairwise registration of the LPBA40 image volumes. As before, pairwise registration is performed. The images with pathologies serve as reference and the lesion-free images as moving images. Since we expect fewer non-correspondences when using the entire image volumes than in the 2D setting, where misalignments between slices can occur, the weighting parameters in the loss function are set to $\alpha =4$, $\beta =2\mathrm {e}^{-2}$, $\gamma =6\mathrm {e}^{-3}$ and $\lambda =1$. In the following, we (1) report registration performance over all 56 labels, (2) compare results to 3D VoxelMorph and (3) report segmentation results for unsupervised and weakly supervised lesion segmentation in 3D.

**Fig. 6:**
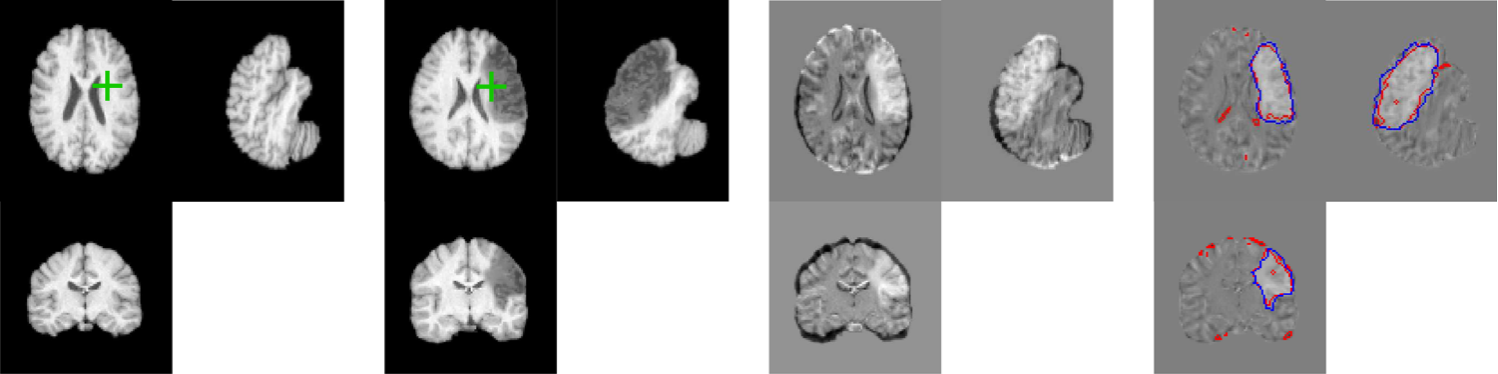
Exemplary 3D result of NCR-Net. Shown are the moving image, the fixed image with lesion L4 and the difference images before and after registration. Green crosses indicate the position of the other depicted image slices. Overlaid onto the difference image after registration the ground truth and generated lesion segmentations are shown in blue and red

Table 4 summarizes the 3D registration results for NCR-Net and VoxelMorph. Given are the mean Jaccard indices and their standard deviation averaged over all 56 labels before and after registration. As in the 2D setting, NCR-Net outperforms VoxelMorph and it can again be observed that the performance of NCR-Net is not much affected by non-correspondences. The robustness of NCR-Net is further confirmed by the values of the standard deviation showing much less variation in the NCR-Net results compared to VoxelMorph. In Fig. 6, an exemplary registration and segmentation result is shown for the 3D NCR-Net. Note that the 3D version of NCR-Net segments less non-correspondent regions as it profits from the entire image information (cf. Fig. 3).

Using the non-correspondence output of NCR-Net to segment the phantom stroke lesions in 3D average DSCs of 0.584, 0.039, 0.028 and 0.679 are achieved for lesions L1 to L4, respectively. With region growing, these can be improved to 0.713, 0.675, 0.071 and 0.760. This shows that large lesions L1 and L4 are again well recognized and outlined, and even the small lesion L2 is segmented well using region growing. Only for lesion L3 results are inferior, which might be due to the comparably high weighting of the segmentation regularization and the fact that the lesion has a rather low contrast to healthy tissue compared to the other lesions.

To measure how good the network’s lesion detection rate is, we calculate the proportion of lesions that did not have a voxel segmented by the network. Both large lesions were detected in each of the 1560 runs performed per lesion type. Lesion L2 was missed only three times and even lesion L3 was detected in $83.65\%$ of the runs. Although the lesion is not well segmented, its detection rate remains high. This could be used, for example, to provide guidance on image examination. Overall, NCR-Net scales well to 3D both for the registration and the non-correspondence detection task.

## Discussion

We presented NCR-Net, a new network architecture for joint image registration and unsupervised non-correspondence segmentation. The network was trained with a two-step training procedure that first pre-trains encoder and registration decoder and later updates the entire network based on a Mumford–Shah-like functional. Here we used rough anatomical labels to introduce weak supervision into the registration task but also showed that NCR-Net may be trained fully unsupervised without significant drop in performance. NCR-Net was additionally shown to profit from the generated non-correspondence maps that prevent implausible deformations in pathological areas.

The resulting deformation fields and segmentations of non-corresponding regions may be used to visualize disease progression in OCT image slices of AMD patients. Based on outlier detection in the image distance measure and without the need for manual segmentations of lesions, NCR-Net learned to segment regions containing altered or newly developed pathologies in OCT images. The lesion segmentation abilities of NCR-Net were quantitatively confirmed using phantom stroke lesions in MR images. This shows great potential of our CNN to be used to generate sharp and detailed segmentations of lesions in an unsupervised manner. In the two-dimensional setting, NCR-Net showed a comparable performance with state-of-the-art registration methods for lesion-free images and surpassed the other methods for images with major pathologies. The three-dimensional extension of NCR-Net outperformed VoxelMorph, one of the state-of-the-art CNN-based registration methods. Overall, the performance of NCR-Net is competitive to state-of-the-art registration methods and robust to a wide variety of lesions thanks to the non-correspondence detection part of the network. The segmentations given by NCR-Net are usable for the segmentation of newly appeared or altered pathologies, the detection of dissolved lesions or the analysis of inter-patient variations with the great advantage that no expensive manual segmentations are needed for training. Our further research will concentrate on improving the separation of spatial deformations and cost function masking even more and making the 3D extension of NCR-Net usable for larger image resolutions.

## Data Availability

The LPBA40 data that support the findings of this study are available from the Laboratory of Neuro Imaging (“LONI”) under the Atlas Distribution Agreement. Due to privacy and ethical concerns, the optical coherence tomography dataset analyzed during the current study cannot be made available.

## Declarations

### Code availability

The source code used to generate the results in this study will be made available on https://github.com/juliaandresen/NCRNet.git.
